# Early Strength Enhancement Mechanism of CaO-Modified Electrolytic Manganese Residue-Based Supersulfate Cement

**DOI:** 10.3390/ma18020270

**Published:** 2025-01-09

**Authors:** Yundan Du, Qing Chen, Fufei Wu, Weiwei Li, Luxian Meng, Yang Liu

**Affiliations:** 1 School of Materials and Architectural Engineering, Guizhou Normal University, Guiyang 550025, China; 460180829@gznu.edu.cn (Y.D.); 201608014@gznu.edu.cn (F.W.); 460022492@gznu.edu.cn (W.L.); 232200191857@gznu.edu.cn (L.M.); 2 Department of Architecture and Civil Engineering, Guizhou Communications Polytechnic University, Guiyang 550025, China; chenqing_gzcpu@163.com; 3 Guizhou Provincial Architectural Design & Research Institute Co., Ltd., Guiyang 550025, China

**Keywords:** electrolytic manganese residue (EMR), CaO-modified EMR-based supersulfate cement (SSC), alkali activator, sulfate, mechanical properties, microanalysis

## Abstract

Electrolytic manganese residue (EMR) is a solid waste generated during the production of electrolytic manganese metal through wet metallurgy, accumulating in large quantities and causing significant environment pollution. Due to its high sulfate content, EMR can be utilized to prepare supersulfate cement when combined with Ground Granulated Blast furnace Slag (GGBS). In this process, GGBS serves as the primary raw material, EMR acts as the sulfate activator, and CaO powder, along with trace amounts of cement, functions as the alkali activator. This results in the preparation of CaO-modified electrolytic manganese residue-based supersulfate cement (Abbreviated as “SSC”), facilitating the harmless and resourceful utilization of EMR. This study aims to determine the optimal dosage of CaO as the alkali activator for GGBS in SSC. A comprehensive analysis was conducted on four groups, including a control group. The mass ratio of EMR, GGBS, and cement in SSC was fixed as 35:60:5, and the optimum mixing ratio of lime powder as an external admixture was investigated through mechanical tests and microscopic experiments. The hydration products and mechanism of the cementitious materials were analyzed using X-ray diffraction (XRD), pH measurements, thermogravimetric and differential thermogravimetric analysis (TG-DTG), mercury intrusion porosimetry (MIP), and scanning electron microscopy (SEM). The results indicated that, under the combined influence of trace cement and raw lime powder, EMR effectively activated GGBS. The primary hydration products of the SSC are AFt and hydrated calcium silicate (C-S-H), which contributed to the mechanical strength of the SSC. At a hydration age of 3 days, the optimal CaO blending ratio was found to be 8% by mass of dried EMR. With this ratio, the compressive strength of SSC reached 18.2 MPa, the pore size of hardened slurry was refined, the structure became dense, and hydration products increased. It could be concluded that CaO enhances the early strength of SSC when used as an alkali activator.

## 1. Introduction

Electrolytic manganese residue (EMR) is an industrial solid waste generated during the filter pressing and purification processes in electrolytic manganese production [1,2]. With the increasing depletion of manganese ore resources and the decline in manganese ore grade in China, 8 to 12 tonnes of EMR are produced for every 1 tonne of electrolytic manganese [3]. By the end of 2022, the stockpile of EMR exceeded 130 million tonnes and is growing at a rate of approximately 10 million tonnes per year [4,5]. The traditional disposal method for electrolysis manganese slag involves open storage or landfill [6]. However, ammonia nitrogen and soluble manganese pollutants from the slag can leach into the soil, rivers, and groundwater through rainfall or improper seepage control measures at disposal sites, thereby damaging the ecological balance and posing risks to human health [7,8]. Therefore, research on the harmlessness and resource utilization of EMR is essential for promoting the comprehensive use of this resource.

EMR is rich in sulfate, which can be used as an activator for GGBS, fly ash, and other potentially active materials with volcanic ash to prepare composite cementitious materials [9]. Currently, most studies suggest the use of EMR for the preparation of composite cementitious materials by first calcining and mechanically grinding EMR [10], followed by its combination with GGBS and other active materials. Wang Zhi et al. [11] prepared a composite cementitious material using quicklime, EMR, fly ash, and cement, achieving a compressive strength reaching 10.5 MPa at 28 days. Xue et al. [12] prepared a composite cementitious material using EMR, lime, cement, and GGBS, with a compressive strength of 6.36 MPa at 28 days. Wang Weizhong et al. [13] prepared a composite cementitious material using EMR, steel slag, red mud, and cement, achieving a compressive strength of 38.6 MPa at 28 days. Li Cheng et al. [14] prepared a composite cementitious material using EMR, GGBS, and slaked lime, reaching a compressive strength of 30.5 MPa at 28 days. He Weilong et al. [15] prepared Belite-calcium sulphoaluminate cement using calcined EMR and barium slag, demonstrating that the carbon emission index of hydration products can be reduced when the dosage of EMR is between 10% and 15%. Wang Fan et al. [16] produced Belite-ye’elimite-ferrite cement at a 1200 °C by utilizing EMR, CaO, and Al_2_O_3_. Wu Zhonghu et al. [17] prepared a geopolymer from calcined EMR, coal slag, and granulated blast furnace slag, with the calcination temperature set at 900 °C. Wang Fan et al. [18] developed electrolytic manganese residue-based supplementary cementitious materials using calcined and ground EMR, GGBS, and fly ash, with EMR dosages ranging from 0% to 20%. All the EMR used in these studies were calcined and ground, which enhances the reactivity of EMR. While this method is resource-efficient for EMR, it involves high energy consumption. Moreover, the doping of EMR is minimal, resulting in the low combined resource utilization of EMR.

In contrast, this study uses uncalcined and unground EMR. The preparation of supersulfate cement (SSC) involves using EMR and GGBS as the main raw materials, blended with trace cement and CaO powder to create a suitable alkaline environment to activate the volcanic ash activity of GGBS. This harmless treatment method neutralizes the acid in EMR and regulates its sulfate composition, thereby stimulating the activity of GGBS to facilitate its decomposition [19]. The approach is simple and energy-efficient and aligns with development goals of energy conservation, emission reduction, and low-carbon environmental protection. The optimum ratio of raw lime powder, under a fixed mass ratio of EMR, GGBS, and cement, is determined through mechanical property tests. The microscopic properties are analyzed through experimental techniques to provide scientific theoretical support for the resourceful use of EMR and other sulfate industrial solid wastes.

## 2. Materials and Methods

### 2.1. Raw Materials

**Figure 1:**
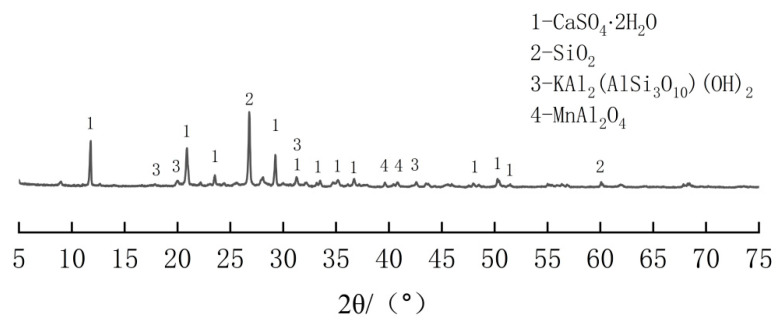
XRD patterns of EMR.

The EMR used in this study was sourced from Songtao Sanhe Manganese Industry Group Limited Liability Company, located in Tongren City, Guizhou Province, China. It was crushed into particles of 1~2 cm size in size, with a moisture content of 24.19% and a pH value of 6.01. The GGBS used was S95 grade slag powder produced by Guiyang Changle Iron and Steel Co. (Guiyang, China). The cement ordinary silicate cement (P·O42.5) was manufactured by Guizhou Zunyi Conch Panjiang (Zunyi, China), with a density of 3.06 g/cm^3^. The alkali activator was raw lime powder, an analytically pure reagent. The main chemical compositions of GGBS and EMR are shown in Table 1, and the XRD pattern of EMR is shown in Figure 1. The mineral composition of EMR is mainly CaSO_4_·2H_2_O and SiO_2_ [20]. The water reducing agent used was a high-efficiency product with a solid content of 25%, produced by Jiangsu Subot New Materials Co. (Nanjing, China).

### 2.2. Sample Preparation

#### 2.2.1. Preparations of CaO + EMR Powders

The raw, wet, crushed EMR slag and CaO powder were weighed according to the four sets of EMR + CaO ratios outlined in Table 2. The CaO dosage in Table 2 represents the mass percentage of the dried EMR slag. The crushed EMR slag was mixed with CaO powder, and 45% of the mass of the dry EMR slag was added as water. The mixture was then placed in a net slurry mixer and blended at low speed for 240 s, followed by a 2 h standing period. Samples of CaO-modified EMR were immersed in anhydrous ethanol for 3 days to terminate hydration. Afterward, the specimens were removed and placed in an oven at 50 °C for 3 days. Once dried, the specimens were ground using an onyx mortar and filtered through a 200-purpose sieve, producing the CaO + EMR powder with a particle size of less than 0.015 mm, which was used for XRD, TG, and pH tests. The associated experiments involving the CaO + EMR powder samples were referred to as “Experiments in the C series”.

#### 2.2.2. Preparations of SSC

Table 3 shows the design of the SSC mix ratio. The mass ratio of EMR, GGBS, and OPC in the SSC was fixed at 35:60:5, with a water to EMR + GGBS + OPC ratio of 0.4. To facilitate specimen casting and molding, water-reducing agents were added to the D3 and D4 groups, with the amount of water-reducing agent expressed as a percentage of the total mass of the cementitious materials (EMR + GGBS + OPC).

**Figure 2:**
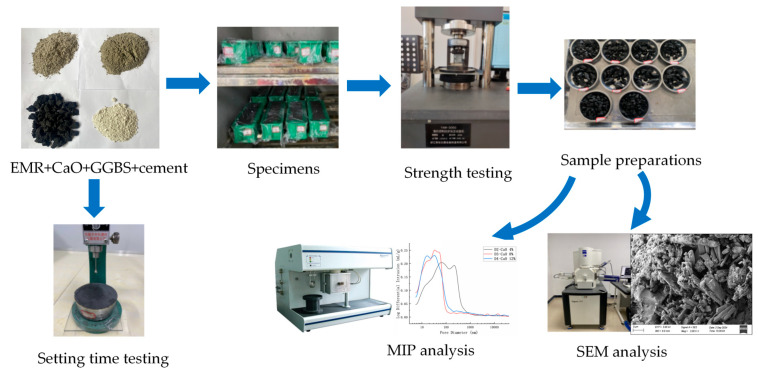
Flowchart of SSC sample preparations and experiments in the D series.

According to the proportion design in Table 3 and following the test method outlined in Method of Testing Cements—Determination of Strength (GB/T 17671-2021) [21], the raw materials for SSC were placed in a net slurry mixer and mixed at low speed for 240 s to ensure homogeneous blending. The 40 mm × 40 mm × 40 mm molds were prepared by brushing oil; the prepared slurry was poured into the molds, which were then vibrated on a vibrating table for 60 s. The molded specimens were placed in the curing box at (20 ± 2) °C and 95% humidity. After 24 h of curing, the specimens were demolded (except for the D1 group with 0% CaO, which was demolded after 72 h) and further cured for 3, 7, 14, and 28 days before undergoing compressive strength tests. Samples from the damaged specimens were immersed in anhydrous ethanol for 3 days to terminate hydration and then used for MIP and SEM tests. The preparation process for SSC samples and the related experiments, referred to as “Experiments in the D series”, is shown in Figure 2.

### 2.3. Test Methods

#### 2.3.1. Physico-Mechanical Testing

The compressive strength test of the hardened slurry of SSC was conducted using the YAW-300C microcomputer-controlled flexural and compressive testing machine produced by Zhejiang Yingsong Instrument and Equipment Manufacturing Co. in Shaoxing, China. According to the test method outlined in GB/T17671-2021 [21], the loading rate for the compressive strength test was 2.4 kN/s. Three samples were tested for each group, with the results representing the average of the three measurements. The strength value of each test specimen should not deviate from the average by more than ±10%.

The pH value of the solid suspension of CaO + EMR powder samples was tested in accordance with the national standard—Standard for Geotechnical Testing Method (GB/T 50123-2019) [22]. A 3 g sample of powder was weighed and then mixed with 30 mL of deionized water. The mixture was stirred for 30 min using a magnetic stirrer and allowed to stand for 2 h. The pH value of the upper clear liquid was then measured using a Leici PHS-25 pH meter, produced in Shanghai, China, which was used to assess the effect of different CaO dosages on the acidic environment of the raw EMR slag.

The initial and final setting times of the slurry were measured according to Chinese standards (GB/T 17671-2021). A Vicat meter was used for the test, which was conducted at temperature of (20 ± 2) °C. Fresh slurry was poured into a standardized truncated conical metal mold of a height of 40 mm. The starting time was defined as the contact time between the raw material (EMR + GGBS + CaO) and H_2_O. The initial setting time was determined when the depth of the test needle reached 4 mm ± 1 mm from the bottom plate. The final setting time was recorded when the circular cutting edge of the test needle could no longer leave traces on the surface of the specimen.

#### 2.3.2. Instrumental Methods

X-ray diffraction analysis (XRD) was performed using a Bruker D2 phaser, manufactured in Ettlingen, Germany, with a Cu Kα radiation source. The scanning angles ranged from 5° to 90°, and the scan rate was 5°/min. Scanning electron microscopy (SEM) was conducted using a ZEISS Sigma300 field emission scanning electron microscope, also produced in Oberkochen, Germany. The mercury intrusion porosimetry test (MIP) was carried out using a Micromeritics AutoPore V 9620 fully automated mercuric pressure tester, made in the Norcross, GA, USA. The thermogravimetric and differential thermogravimetric (TG-DTG) analysis was conducted using a Hitachi STA7300 thermogravimetric analyzer, produced in Tokyo, Japan. The temperature range for the TG analysis was 30 to 1000 °C, with a heating rate of 10 °C/min.

## 3. Results and Discussion

### 3.1. XRD of C Series

**Figure 3:**
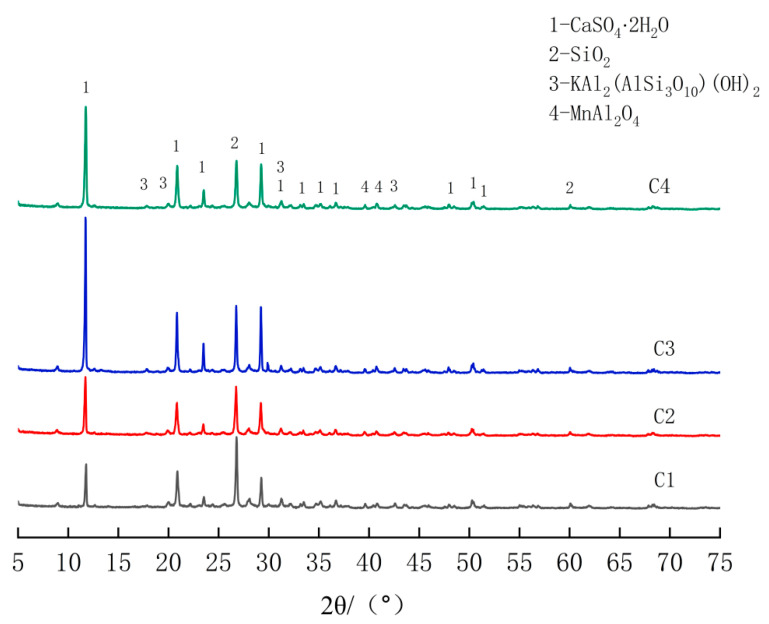
XRD pattern of CaO + EMR.

From the Figure 3, it is evident that the mineral composition of both the EMR and the CaO-modified EMR primarily consists of CaSO_4_·2H_2_O and SiO_2_. The peak intensities at 11.7°, 20.8°, and 29.2° for the CaO-doped test groups were higher than those of the C1 group, which was not doped with CaO. Notably, the C3 group (8% CaO) and the C4 group (12% CaO) exhibited significantly higher peak intensities at these angles, suggesting that CaO doping promotes the formation of more CaSO_4_·2H_2_O in the EMR. This indicates that the higher sulfate content in the EMR enhances the activation of the mineral powder. During hydration, soluble SO_4_^2^⁻ interacts with Ca^2^⁺ and Al^3^⁺ in the slag to form calcium alunite crystals. The increased formation of hydration products in the SSC contributes to the development of strength.

### 3.2. pH of C Series

The pH value of the solid suspension of CaO + EMR powder with different dosages of CaO was 6.01, 8.75, 11.57, and 12.29. These results indicated that the raw EMR slag was acidic and unable to provide a proper hardening environment for the SSC. After the addition of CaO, the solid suspension became alkaline, creating a favorable environment for hydration. This change disrupted the vitreous structure of mineral powders and increased their reactivity. Furthermore, a higher CaO dosage resulted in a greater increase in alkalinity. The pH values for the groups with 8% and 12% CaO doping were similar. The concentration of Ca^2+^ and OH^−^ in the solid suspension increased with the adding of CaO. When the concentrations reached a certain critical value, the hydration products C-S-H and Ca(OH)_2_ began to crystallize and grow, which enhanced the activation of the slag’s volcanic ash activity due to the formation of Ca(OH)_2_ and the presence of gypsum [23]. Ca(OH)_2_ acts as an alkali activator, dissociating the silica-oxygen mesh structure on the surface of the slag vitreous and generating more C-S-H gels. In this alkaline environment, the C-S-H gels remained stable [24].

### 3.3. TG-DTG of C Series

**Figure 4:**
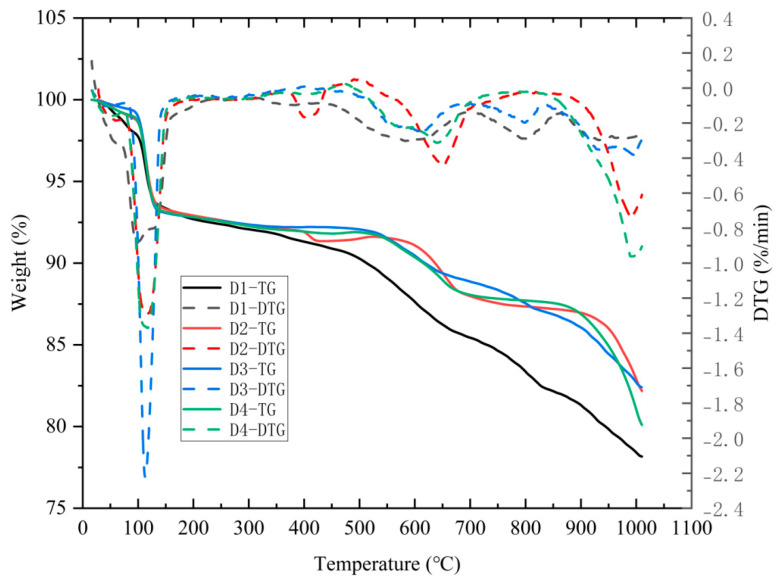
TG-DTG curves of CaO + EMR.

Figure 4 shows the TG-DTG curves of four groups of modified EMR with different CaO dosages, according to the mix ratio design in Table 2. The first weight loss range, from 70 °C to 200 °C, corresponds to the dehydration of CaSO_4_·2H_2_O. The TG-DTG curves show that the weight loss ratios for groups C2, C3, and C4 were higher than that for group C1, indicating that the CaO-modified EMR generated more CaSO_4_·2H_2_O. The 8% and 12% CaO doping groups had a more significant effects in the early stage, providing more SO_4_^2−^ for the early hydration of the SSC and thus facilitating the formation of early strength. The second weight loss range, from 460 °C to 650 °C, corresponds to the decomposition and dehydration of Ca(OH)_2_ [25]. Some of the Ca(OH)_2_ generated from the CaO doping did not react with other compounds and underwent thermal decomposition during this stage. The third weight loss range, around 900 °C, corresponds to the decomposition of CaCO_3_ [26], which may be attributed the carbonization of the sample during the drying and preparation processes.

### 3.4. Setting Time of D Series

According to the ratio design in Table 3, the SSC slurry was prepared. The setting time of the SSC was tested following the method outlined in Method of Testing Cements—Determination of Strength (GB/T 17671-2021), and the results are presented in Table 4. As the CaO dosage increased, both the initial and final setting times of the SSC were shortened, indicating an acceleration of the hydration process and earlier strength development. The setting and hardening processes for the D3 and D4 groups were similar. In contrast, the D1 group, which was not doped with CaO, exhibited a significantly longer setting time due to the poor hydration environment.

### 3.5. Compressive Strength of D Series

**Figure 5:**
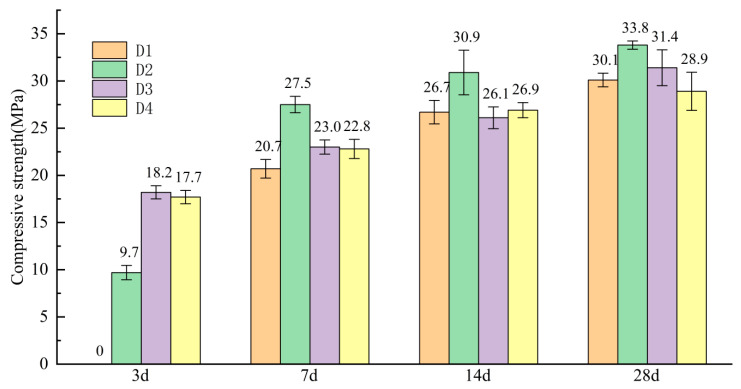
Compressive strength of SSC.

According to the mix ratio design in Table 3, the net slurry specimens of SSC were prepared. The effects of CaO doping on the compressive strength of SSC at different ages were investigated through mechanical property tests. Figure 5 shows the compressive strength of four groups of hardened slurry specimens with different CaO dosages at 3, 7, 14, and 28 days. As shown in Figure 5, the compressive strengths of the specimens in each group increased over time, as hydration products gradually diffused and precipitated. The strengths reached their peak at 28 days. Among them, the D2 group (4% CaO) exhibited the highest compressive strength at 7, 14, and 28 days, reaching 33.8 MPa at 28 days. The other three groups showed slightly lower strengths, but the difference was not significant, suggesting that the optimal dosage of CaO had a slight effect on the late-stage (28-day) strength. Notably, the compressive strength of the D1 group (0% CaO) was slightly higher than that of D4 group (12% CaO), indicating that excessive CaO doping negatively impacted the late-stage strength, leading to a loose structure and reduced strength.

**Figure 6:**
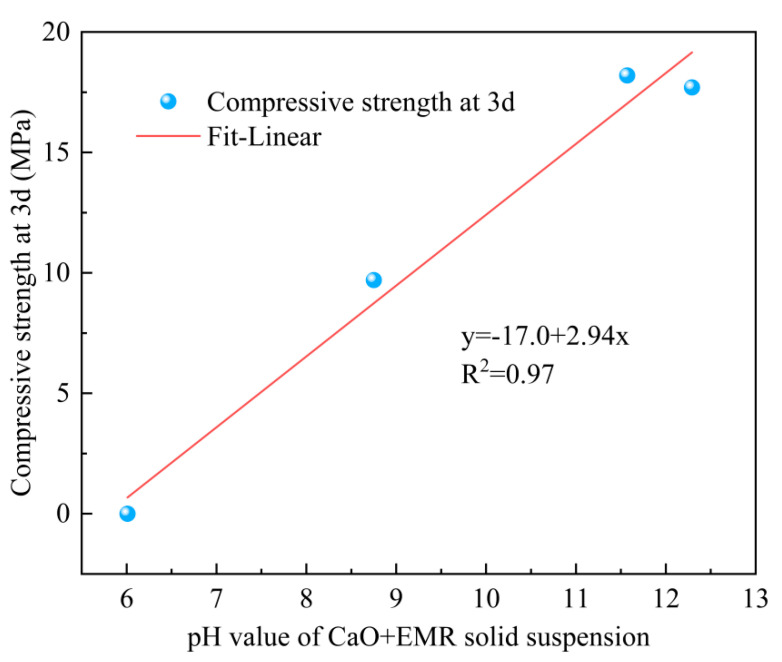
Linear fit of compressive strength and pH value.

In the 3-day compressive strength test, the D3 group showed the highest strength, reaching 18.2 MPa, followed by the D4 group at 17.7 MPa, which was similar to the D3 group. Both were significantly higher than the D2 and D1 groups. Notably, the 3-day compressive strength of the D1 group was 0, indicating that a reasonable amount of CaO significantly contributed to the early strength of the SSC. When CaO reacted with water, it generated Ca(OH)_2_, which improved the initially acidic environment of EMR, making the system alkaline and providing additional Ca^2+^. The strong alkali environment accelerated the breakdown of silica-oxygen and aluminum-oxygen bonds, disrupting the vitreous surface of the mineral powder, and enhancing the volcanic ash activation. This in turn accelerated the early hydration reaction, leading to the formation of the strength at an earlier time [27]. As is shown in Figure 6, there is a linear correlation between the compressive strength of SSC at 3 days and the pH value of the EMR + CaO mixture. This indicates that during the early hydration period, the incorporation of CaO significantly contributed to the early strength development of the SSC.

### 3.6. MIP of D Series

**Figure 7:**
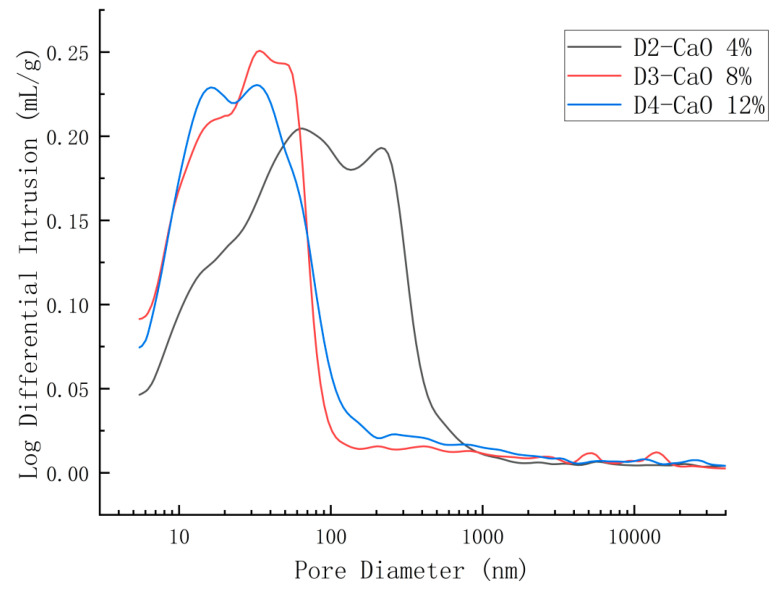
Differential curves of pore size distribution at 3 d.

**Figure 8:**
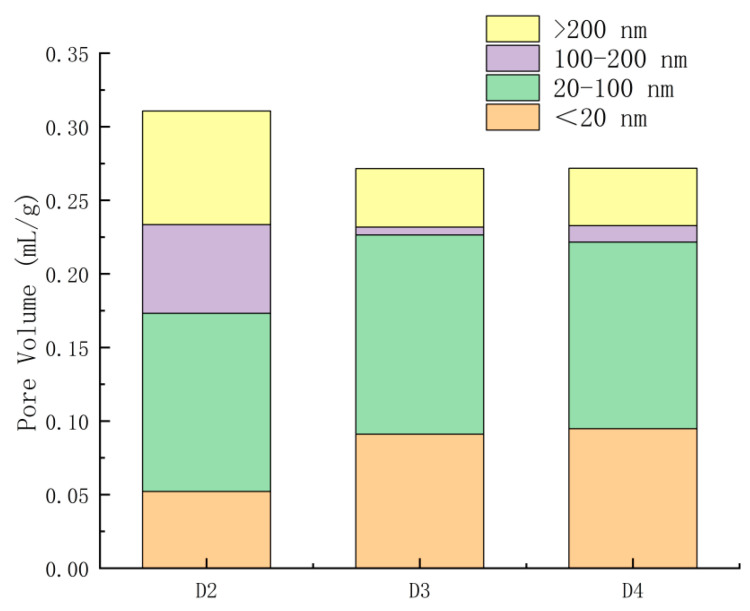
Stacked histograms of pore size distribution at 3 d.

**Figure 9:**
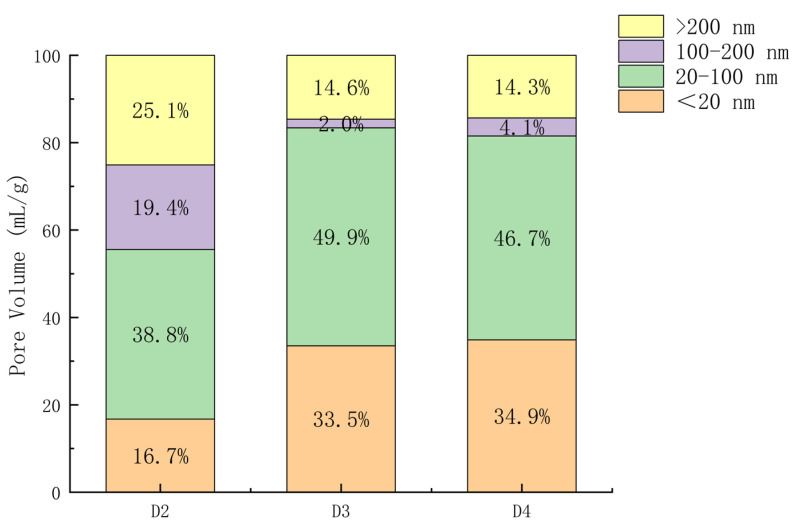
Distribution percentage stacked histograms of pore size distribution at 3 d.

Pore structure is a key structural characteristic of SSC hardened slurry, and the development of its pore size distribution provides insight into the degree of the reaction of SSC. The differential curves of pore size distribution for SSC hardened slurry at 3 days are shown in Figure 7, while the stacked histograms of pore size distribution are shown in Figure 8 and the distribution percentage histograms are shown in Figure 9. Since the compressive strength of the D1 group at 3 days was 0, the MIP test was conducted on only three groups: D2, D3, and D4.

Based on the effects of different pore sizes on the properties of concrete, Zhongwei Wu classified pores into four levels: harmless pores (pore size < 20 nm), less harmful pores (20 nm < pore size < 100 nm), harmful pores (100 nm < pore size < 200 nm), and multi-harmful pores (pore size > 200 nm) [28]. As shown in Figure 8, the total pore volume of the groups D3 and D4 was significantly reduced compared with group D2 (4% CaO doping), with both harmful pores (100 nm < pore size < 200 nm) and multi-harmful pores (pore size > 200 nm) being notably decreased. As illustrated in Figure 9, the volume of harmless pores was approximately twice as large in groups D3 and D4 as in group D2. The volume of harmful and multi-harmful pores was considerably lower in group D3, at 38% of that in group D2, and in group D4 at 40% of that in group D2. From Figure 7, it can be observed that the most available pore diameters of groups D2, D3, and D4 were 64.11 nm, 33.92 nm, and 32.97 nm, respectively. The most available pore diameters decreased as CaO doping increased, with the diameters of groups D3 and D4 being very similar. CaO promoted the early hydration of SSC. With a moderate increase in CaO dosage, more Ca^2+^ and (OH)^−^ were provided, facilitating the depolymerization of the slag vitreous structure and generating more hydration products to fill the pore space. This densified the slurry structure, refined the pore size, reduced the pore volume, and strengthened the bond at the aggregate interface [29,30]. At an 8% CaO dosage, a reasonable dosage was achieved, with the pore size distribution changes and compressive strength performance results maintaining consistency.

### 3.7. SEM of D Series

**Figure 10:**
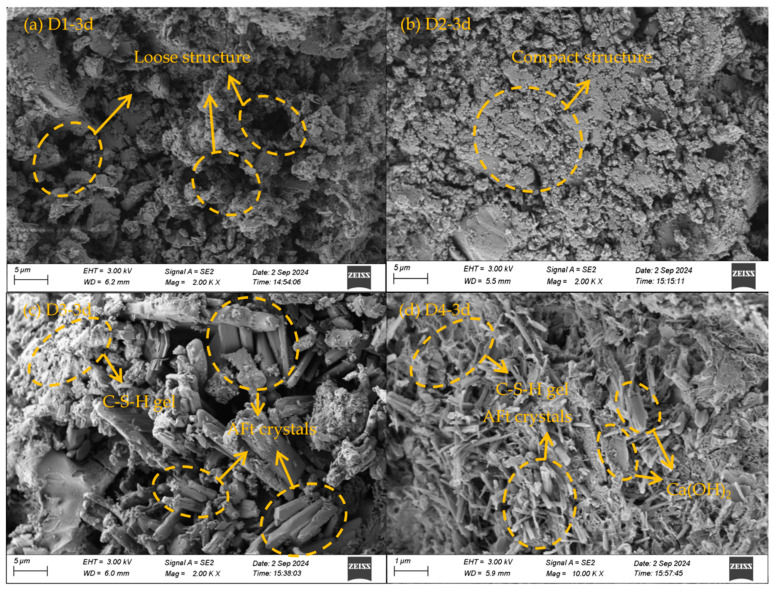
SEM images of SSC paste hardener at 3 d.

The SEM morphologies of SSC net mortar specimens with different CaO dosages at 3 days of curing are shown in Figure 10. As can be seen from Figure 10a, the specimen without CaO exhibited a loose structure and large porosity at 3 days, with no visible hydration products, indicating that strength formation had not yet occurred. In Figure 10b, with 4% CaO doping, some hydration products, including C-S-H gels, were observed. The structure became more compact, and the porosity between the particles decreased, providing early-stage strength support. In Figure 10c, with 8% CaO doping, a significant amount of the hydration products, primarily AFt and C-S-H gel, are seen to have formed. The needle-like AFt crystals are interspersed between different particles, intertwining with the C-S-H gel. As the AFt crystals continued to grow, the C-S-H gel filled the gaps, reducing pore space and densifying the structure, leading to a substantial improvement in the specimen’s strength. In Figure 10d, with 12% CaO doping, the structure is seen to be compact, with large quantities of AFt crystals and C-S-H gel formed and interwoven. Some flakes of Ca(OH)_2_ are also visible. The C-S-H gel surrounded the particles and AFt crystals, further densifying the structure, resulting in higher compressive strength, consistent with the macro-mechanical property test results.

### 3.8. Hydration Mechanism of D Series

Comprehensive macroscopic and microscopic test analyses showed that in the SSC system, the activity of the GGBS was effectively activated during the hydration process under the dual excitation of sulfate and alkali activators. The physical phase of GGBS is primarily vitreous. During the prehydration period, the active Al_2_O_3_ and SiO_2_ in the GGBS provided the silica-aluminum component of the system, while the EMR supplied abundant sulfate. A small amount of cement and CaO created a strong alkaline environment conducive to the hydration reaction. In the alkaline solution state, the high concentration of (OH)^−^ caused the Si-O-Si and Al-O-Al bonds to break, releasing (SiO_3_)^2−^ and (AlO_4_)^5^, which then reacted chemically with Ca^2+^ to form gel substances such as C-S-H, providing strength support to the system [31,32]. Unlike ordinary Portland cement systems, the hydration process in SSC is illustrated in Equation (1) [33,34].

(1) $$C_{5}S_{3}A+CH+3C\bar{S}+34H\to C_{6}A{\bar{S}}_{3}H_{32}+3CSH$$

C_5_S_3_A is the average composition of GGBS. The phase is amorphous and therefore requires an activator to react. The presence of gypsum in the EMR allows a sulfato-calcic activation, the formation of C-(A)-S-H and ettringite (AFt) from gypsum and lime [35,36,37,38]. As the hydration reaction proceeded, the growth of AFt consumed a large amount of SO_4_^2−^. Some SiO_3_^2−^ in the C-S-H gel was replaced by SO_4_^2−^, becoming free SiO_3_^2−^ ions, which in turn reacted with free Ca^2+^ to generate more C-S-H gel [14], thereby further enhancing the hydration reaction. The strength of the hardened SSC slurry primarily is derived from the C-S-H gel and AFt. The flocculated C-S-H gel and needle-like AFt crystals were interspersed with each other, and as the hydration age increased, the volume of hydration products in the hardened slurry increased, leading to a denser internal structure and greater strength.

## 4. Conclusions

In this study, the uncalcined and unground EMR slag was used as the research object to analyze the microscopic properties of CaO-modified EMR as well as the mechanical properties and microscopic properties of SSC. The following conclusions were drawn:

(1) When the mass ratio of the EMR–GGBS–cement was fixed as 35:60:5 and the dosage of external dopant CaO was 8%, the compressive strength of the cementitious system was maximized at 3 days, reaching 18.2 MPa. At 4% CaO doping, the compressive strengths at 7, 14, and 28 days reached 27.5 MPa, 30.9 MPa, and 33.8 MPa, respectively.
(2) The addition of CaO promoted the formation of more gypsum dihydrate, which provided an increased amount of SO_4_^2−^. The effect was most pronounced at a CaO dosage of 8%, improving the acid-base environment and thereby accelerating the hydration reaction.
(3) As the CaO doping increased, the setting time of SSC was progressively shortened. The setting times of the 8% and 12% CaO-doped systems were very similar. The increased CaO content accelerated the hydration rate, which facilitated the earlier formation of strength.
(4) The porosity of the hardened slurry gradually decreased with moderate increases in CaO doping. The pore size was refined, with a higher proportion of harmless and less harmful pores, leading to a densified slurry structure. The optimal pore size distribution was observed at 8% CaO doping after 3 days. Flaky Ca(OH)_2_ crystals were found in the specimen with 12% CaO doping.
(5) The main hydration products of SSC were C-S-H gel and AFt. The alkaline environment provided by CaO and trace amounts of cement activated the SO_4_^2−^ in EMR, enhancing the reactivity of GGBS. This accelerated the hydration process and contributed to the strength development of the system.

In conclusion, CaO serves as an effective alkali activator, significantly enhancing the early strength of SSC. The EMR used in this study does not require calcination or milling. The approach is easy to implement and energy-efficient, which will contribute to enhancing the harmlessness and resource utilization of EMR.

## Figures and Tables

**Table 1:** Main chemical compositions of EMR, cement, and GGBS (wt.%).

Chemical Compositions	SiO_2_	SO_3_	Al_2_O_3_	CaO	Fe_2_O_3_	K_2_O	MnO	MgO	Na_2_O	P_2_O_5_	TiO_2_
EMR	39.77	20.48	13.88	9.19	6.24	3.47	2.92	1.59	1.05	0.74	0.51
GGBS	26.37	3.16	14.27	40.26	0.80	0.88	1.78	9.07	0.81	0.03	2.11
cement	19.72	2.77	5.76	58.01	4.01	1.26	0.43	2.03	0.42	0.15	0.24

**Table 2:** Material ratios of EMR + CaO.

No.	EMR Dosage	Water Dosage %	CaO Dosage %
C1	1	45	0
C2	1	45	4
C3	1	45	8
C4	1	45	12

**Table 3:** Material ratios of SSC (wt%).

No.	EMR Dosage	GGBS Dosage	OPC Dosage	Water-Binder Ratio	Dosage of CaO	Water-Reducing Agent
D1	35	60	5	0.4	0	0
D2	35	60	5	0.4	4	0
D3	35	60	5	0.4	8	0.1
D4	35	60	5	0.4	12	0.17

**Table 4:** Setting time of SSC.

No.	EMR Dosage/%	GGBS Dosage/%	OPC Dosage/%	CaO Dosage/%	Initial Setting Time/h	Final Setting Time/h
D1	35	60	5	0	34	42
D2	35	60	5	4	23.5	32
D3	35	60	5	8	15	24.5
D4	35	60	5	12	14	22.5

## Data Availability

The original contributions presented in this study are included in the article. Further inquiries can be directed to the corresponding author.

## References

[1] Wang D.Q., Fang J.R., Wang Q., Liu Y.J. (2022). Utilizing desulphurized electrolytic-manganese residue as a mineral admixture: A feasibility study. Cem. Concr. Compos..

[2] Wang D.Q., Wang Q., Xue J.F. (2020). Reuse of hazardous electrolytic manganese residue: Detailed leaching characterization and novel application as a cementitious material. Resour. Conserv. Recycl..

[3] Fu T., Pang B., Li H.X., Liang J.L., Bao H.M. (2020). Electrolytic Manganese Residue-Modified Asphalt Performance Test and Micromechanism Analysis. Adv. Mater. Sci. Eng..

[4] Su H.L., Zhou W.T., Lv X.J., Liu X., Gao W.H., Li C.M., Li S.H. (2023). Remediation treatment and resource utilization trends of electrolytic manganese residue. Miner. Eng..

[5] Wang F., Long G.C., Ma K.L., Zeng X.H., Tang Z., Dong R.Z., He J.H., Shangguan M.H., Hu Q.C., Liew R.K. (2023). Recyling manganese-rich electrolytic residues review. Environ. Chem. Lett..

[6] Shu J.C., Wu H.P., Chen M.J., Wei L., Wang B., Li B., Liu R.L., Liu Z.H. (2019). Simultaneous optimizing removal of manganese and ammonia nitrogen from electrolytic metal manganese residue leachate using chemical equilibrium model. Ecotoxicol. Environ. Saf..

[7] Lv Y., Li J., Ye H.P., Du D.Y., Li J.X., Sun P., Ma M.Y., Wen J.X. (2019). Bioleaching behaviors of silicon and metals in electrolytic manganese residue using silicate bacteria. J. Clean. Prod..

[8] Fu X.X., Xiao X., Tan D.Y., Xu Z.H., Yu W.B. (2024). Heavy metal pollution of soils around an electrolytic manganese waste residue storage: Characteristics and evaluation. Environ. Sci. Technol..

[9] Cai Y.X., Long G.C., Xiao Q.Y., Ma K.L., Zeng X.H., Tang Z., Wang J.L. (2023). Effect of low temperature calcined electrolytic manganese residue on the early-age hydration of cement paste. Constr. Build. Mater..

[10] Wang J., Peng B., Chai L., Zhang Q., Liu Q. (2013). Preparation of electrolytic manganese residueground granulated blastfurnace slag cement. Powder Technol..

[11] Wang Z., Gao C.C., Wang Q.Z. (2013). Preparation of electrolytic manganese residue composite cementing material. Non-Met. Mines.

[12] Xue F., Wang T., Zhou M. (2020). Self-solidification/stabilisation of electrolytic manganese residue: Mechanistic insights. Constr. Build. Mater..

[13] Wang Z.W., Chen P., Zhou H.Y. (2019). Study on the heat of hydration of steel slag manganese slag red mud composite cementitious material. Non-Met. Mines.

[14] Li C., Xu Y., Zhu W.Y., Li Z.L., Peng B., Li Y.B., He G.X. (2024). Preparation and Hydration Mechanism of Modified Electrolytic Manganese Slag-GGBS Composite Cementitious Materials. Nonferrous Met. (Extr. Metall.).

[15] He W.L., Li R., Nie D.P., Zhang J., Wang Y., Zhang Y., Chen Q.L. (2022). Belite-calcium sulphoaluminate cement prepared by EMR and BS: Hydration characteristics and microstructure evolution behavior. Construct. Build. Mater..

[16] Wang F., Long G.C., Bai M., Shi Y.Y., Zhou J.L. (2023). A new perspective on Belite-ye’elimite-ferrite cement manufactured from electrolytic manganese residue: Production, properties, and environmental analysis. Cem. Concr. Res..

[17] Wu Z.H., Zhang H., Pu S.Y., Cai G.J., Duan W., Song H.L., Zeng C., Yang Y.H. (2024). Synergistic preparation of geopolymer using electrolytic manganese residue, coal slag and granulated blast furnace slag. J. Build. Eng..

[18] Wang F., Long G.C., Bai M., Shi Y.Y., Zhou J.L. (2023). Feasibility of low-carbon electrolytic manganese residue-based supplementary cementitious materials. Sci. Total Environ..

[19] Erdem E., Olmez H. (1993). The mechanical-properties of supersulphated cement containing phosphogypsum. Cem. Concr. Res..

[20] Xiao Q.Y., Cai Y.X., Yu X., Wang J.L., Ma K.L., Zeng X.H., Tang Z., Long G.C. (2023). Regulating the early age hydration of cement-solidified electrolytic manganese residues paste by alternating current. Constr. Build. Mater..

[21] (2021). Method of Testing Cements-Determination of Strength.

[22] (2019). Standard for Geotechnical Testing Method.

[23] Kolani B., Buffo-Lacarrière L., Sellier A., Escadeillas G., Boutillon L., Linger L. (2012). Hydration of slag-blended cements. Cem. Concr. Compos..

[24] Han F.H., Zhang Z.Q. (2024). Solid Waste-Based Supplementary Cementitious Materials.

[25] Wu M., Zhang Y.S., Jia Y.T., She W., Liu G.J., Yang Z.Q., Zhang Y., Zhang W.T., Sun W. (2019). Effects of sodium sulfate on the hydration and properties of lime-based low carbon cementitious materials. J. Clean. Prod..

[26] Long L., Zhao Y.M., Lv G.J., Duan Y., Liu X.B., Jiang X.G. (2023). Improving stabilization/solidification of MSWI fly ash with coal gangue based geopolymer via increasing active calcium content. Sci. Total Environ..

[27] Wang F., Long G., Zhou J.L. (2023). Deep insight into green remediation and hazard-free disposal of electrolytic manganese residue-based cementitious material. Sci. Total Environ..

[28] Wu Z.W., Lian H.Z. (1999). High Performance Concrete.

[29] Chen R.F., Chen C.L., Yang H.S., Zhao Z.H., Liu X.Y. (2022). Effect of phosphorus slag powder replacing fly ash on performance of MgO-admixed RCC. Adv. Sci. Technol. Water Resour..

[30] Luo W.J., Li B., Yang G., Xu M.X., Pang C.H., Kou K.W., Wu T. (2024). Utilisation of electrolytic manganese residue as a sulphate activator in producing concrete blocks with high-volume fly ash. J. Clean. Prod..

[31] Peng Q.H., Cao D.W., Wan T.L., Leng Z., Tang L. (2023). Mechanical and Microscopic Properties of Cementitious Material with NaOH-Electrolytic Manganese Residue-Slag. J. Highw. Transp. Res. Dev..

[32] Shi M.G., Ke G.J., Zou P.Y., Song B.X., Tang X.L., Jin D. (2022). Research progress of hydration, mechanical and dry shrinkage properties of alkali-activated slag cement. Bull. Chin. Ceram. Soc..

[33] Gracioli B., Luz C., Beutler C.S., Filho J.I.P., Hooton R.D. (2020). Influence of the calcination temperature of phosphogypsum on the performance of supersulfated cements. Constr. Build. Mater..

[34] Angulski D.L., Hooton R.D. (2015). Influence of curing temperature on the process of hydration of supersulfated cements at early age. Cem. Concr. Res..

[35] Bonnet J., Mosser-Ruck R., Sterpenich J., Bourdelle F., Verron H., Michau N., Bourbon X., Linard Y. (2022). Chemical and mineralogical characterizations of a low-pH cementitious material designed for the disposal cell of the high-level radioactive waste (HLW). Cem. Concr. Res..

[36] Ren P.C., Zheng H.P., Jin Z.Q., Li M.Y., Li J.X., Pang B. (2023). Transformation mechanism of AFt and AFm in geothermal environment. Bull. Chin. Ceram. Soc..

[37] Zhou J.W., Yu B.U., Kong Y.N., Yang W., Cheng B.J., Wu J. (2022). Effect of calcium hydroxide on the microstructure and performance of super sulfated cement. Ceram.-Silikáty.

[38] Yu B.Y., Gao Y.X., Wang J. (2014). Hydration behavior of super sulphated cement with different types of gypsum. J. Build. Mater..

